# Thermomechanical Fatigue Testing on Fe-Mn-Si Shape Memory Alloys in Prestress Conditions

**DOI:** 10.3390/ma16010237

**Published:** 2022-12-27

**Authors:** Eva Marinopoulou, Konstantinos Katakalos

**Affiliations:** Laboratory of Strength of Materials and Structures, Department of Civil Engineering, Faculty of Engineering, Aristotle University of Thessaloniki, 541 24 Thessaloniki, Greece

**Keywords:** ferrous shape memory alloys, prestress, recovery stress, relaxation, thermomechanical behavior, fatigue, active materials, low-cost SMAs, civil engineering applications

## Abstract

Active materials have gained increasing momentum during the last decades due to their ability to act as sensors and actuators without the need for an external controlling system or an electronic signal. Shape memory alloys (SMAs), which are a subcategory of active materials, are slowly being introduced in the civil engineering sector in applications that refer to prestressing and strengthening of various structural elements. Low-cost iron-based SMAs are a good alternative to the Ni-Ti SMAs for such uses since the cost of large-scale civil engineering applications would otherwise be prohibitive. The scope of this study is the investigation of the thermomechanical response of the Fe-17Mn-5Si-10Cr-4Ni-1(V,C) ferrous SMA. In particular, this study focuses on the application of prestress, and on the alloy’s behavior under fatigue loadings. In addition, the effect of loading frequency on the recovery stress of the material is thoroughly investigated. Four dog-bone specimens were prepared and tested in low-cycle fatigue. All the experiments aimed at the simulation of prestress. The recovery stress was monitored after pre-straining and heating applied under strain–control conditions. The experimental results are promising in terms of the is situ prestress feasibility since the measured recovery stress values are satisfactory high.

## 1. Introduction

According to Lagoudas [1], shape memory alloys (SMAs) are a unique class of shape memory materials with the ability to recover their shape when the temperature is increased or, under specific conditions, upon cyclic loading. SMAs are categorized as active materials due to their ability to recover strain under stress. Other active materials include piezoelectric and magnetostrictive materials [2]. Due to these attributes, SMAs have been employed as actuators in multiple fields, such as the aerospace and automotive industry; however, they have also been used for biomedical applications, for instance, in orthodontic wires. Ni-Ti alloys are the most popular in these fields. However, their cost is restrictive for civil engineering applications. Iron-based SMAs are an alternative to this issue. Some civil engineering applications include prestressing, vibration dampening, strengthening and actuation.

### 1.1. Shape Memory Effect

Shape recovery takes place upon transformation between two distinct crystal phases that exist on the microstructure of SMAs: austenite and martensite. These two phases differ in both properties and microstructure.

Austenite has typically a cubic structure, while martensite has a structure of lower symmetry. Martensitic transformation is a diffusionless and displacive first-order phase transition driven by stress and/or temperature. The transformation from austenite to martensite is called forward, while the opposite from martensite to austenite is known as reverse transformation.

**Figure 1:**
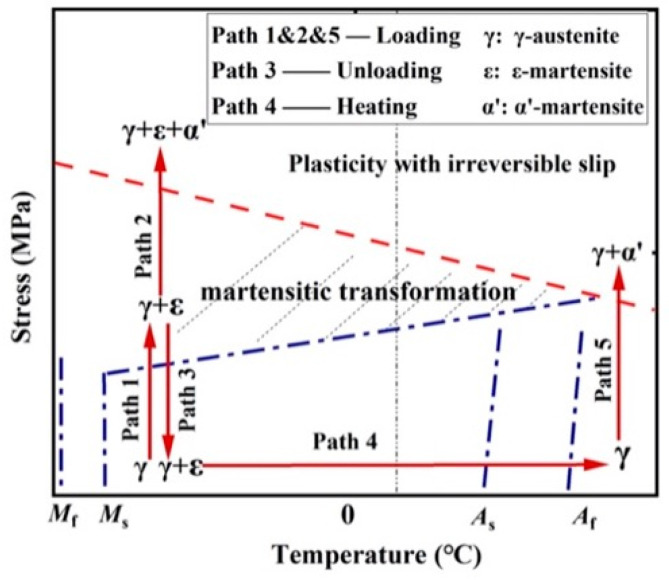
Stress–temperature diagram for an iron-based SMA, Zhang et al. [4].

Martensite is present between Mf and Ms, which are typically low, while austenite can be found between As and Af, which are relatively higher temperatures. These four temperature thresholds create the thermal boundaries of the two phases. Martensite formation can be caused by either thermal or mechanical loading. Thus, it can be categorized into two groups, thermal martensite and stress-induced martensite (SIM), respectively [3]. The phase transformation process during monotonic loading is presented in Figure 1, as obtained from Zhang et al. [4].

### 1.2. Brief Introduction to the Iron-Based SMAs (FeSMAs)

Since Sato et al. discovered that a small Si addition to the chemical composition of an Fe-Mn alloy enhances the shape memory effect [5,6], many iron-based alloys have been developed. Various chemical compositions have been formed, such as Fe-32Mn-6Si by Murakami et al. [7], Fe-28-Mn-6Si-5Cr, Fe-20-Mn-5Si-8Cr-5Ni and Fe-16-Mn-5Si-12Cr-5Ni by Otsuka et al. [8,9].

Tsuzaki et al. [10] proved that adding carbon on an Fe-Mn-Si system strongly enhances the shape memory effect. Many researchers have also demonstrated that austenite can be strengthened through the addition of microparticles, such as niobium and nitrogen [11,12,13]. The shape recovery percentage of iron-based SMAs is significantly affected by both the addition of carbon and the subsequent aging process, as Wen et al. [14] and Farjami et al. [15] indicate.

Aside from these additions in the chemical composition of the alloys, thermomechanical training is also employed to achieve an optimal shape memory effect. The main essence of training an alloy, through various thermomechanical processes, is the enhancement of its mechanical properties. Otsuka et al. [16,17] introduced the term “training” and many researchers continued to examine the topic, proving that these thermomechanical sequences vastly expand the alloys’ shape memory potential [18,19,20,21].

These are all very significant findings that have been thoroughly investigated throughout the years and have made the industrial production of iron-based SMAs possible.

### 1.3. Fatigue Behavior of Iron-Based SMAs under Mechanical Loading

According to Zhang et al. [4], Sawaguchi et al. [22,23] were the first to discover the material’s energy dissipation prospects in 2006. Following these findings, Nikulin et al. [24] discovered that chemical composition plays a major role in fatigue life. It was then demonstrated that silicon presence has a significant impact on fatigue life [25]. In 2021, Fang et al. [26] investigated iron-based SMAs in low-cycle fatigue for strain rates that ranged from ±1% to ±9% and proved that the alloys’ behavior under fatigue could profoundly outperform the steel alloys’ response [26].

A significant amount of research has been conducted since 2006 referring to Fe-Mn-Si systems’ fatigue response in mechanical loading and energy dissipation potential. Among many researchers, particularly Nikulin, Sawaguchi et al. [27,28,29,30], have covered a wide variety of topics, which include microstructural investigations, strain amplitude effect, deformation temperature effect, composition percentages impacts and many more. A comprehensive literature review that covers many studies on fatigue is provided by Zhang et al. [4]. Nonetheless, thermomechanical tests and phase transformation sequences have not been extensively investigated on iron-based SMAs. The present study caters to this exact need, attempting to add more material to the existing literature referring to the implications of low-cycle fatigue on the recovery stress that is required during prestress applications.

### 1.4. Civil Engineering Applications

Iron-based SMAs are used in civil engineering applications during the past decade, in uses relevant to prestressing and strengthening; however, they are also used as energy-dissipating components in seismic damping [26]. Recent advances in additive manufacturing, generally regarding SMAs [31] and particularly focusing on iron-based SMAs [32], could enhance the material’s response and facilitate its incorporation on the field.

In 2016, Shahverdi et al. [33,34] investigated the behavior of concrete beams reinforced with prestressed iron-based SMAs. Following this investigation, further research has confirmed the alloys’ successful use as a prestressing or strengthening component [35,36,37]. On the topic of finite element modeling, few researchers have dealt with the structural simulation of iron-based SMAs. Although there has been extensive investigation on modeling NiTi SMAs, these constitutive models are not quite applicable on Fe-SMAs, since their thermomechanical behavior differs substantially. Khalil et al. [38] have developed a constitutive model, based on previous works of the relevant literature, that described Fe-SMAs’ nonlinearities and phase transformation process, which was also implemented into Abaqus software [38]. Following this attempt, some researchers further examined the material’s modeling towards finite element structural analysis, among which are Abouali et al. [39], who modeled the behavior of prestressed Fe-SMAs used as a reinforcement in a concrete structural element.

## 2. Materials and Methods

**Figure 2:**
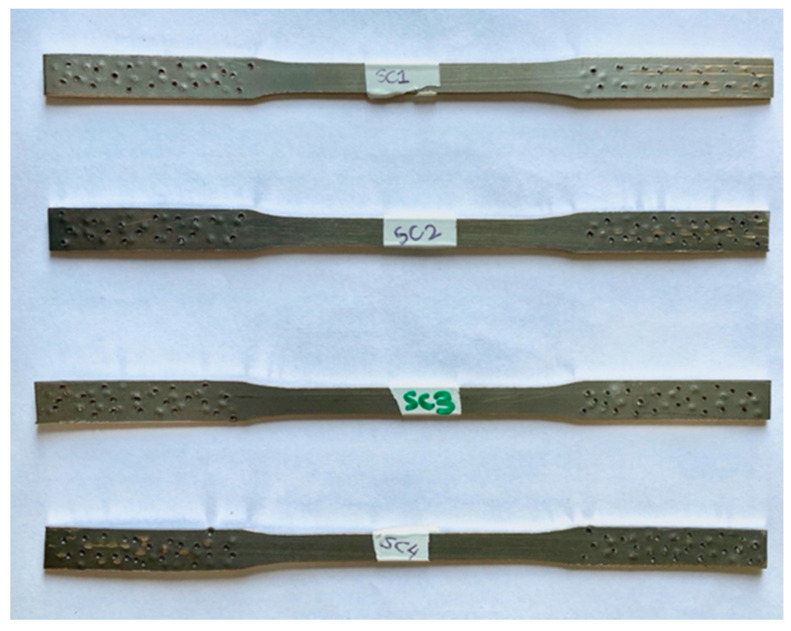
Dog-bone specimens used for the fatigue tests.

**Figure 3:**
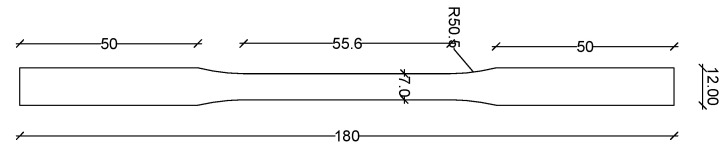
Specimen dimensions (mm).

The iron-based shape memory alloy that was used for this study is the Fe-17Mn-5Si-10Cr-4Ni-1(V,C) (mass%), as-received, without any additional treatments or thermomechanical training. The specimen geometry was chosen to be of dog-bone shape, and the dimensions are displayed in Figure 2 and Figure 3. All the specimens were cut from an SMA strip.

**Figure 4:**
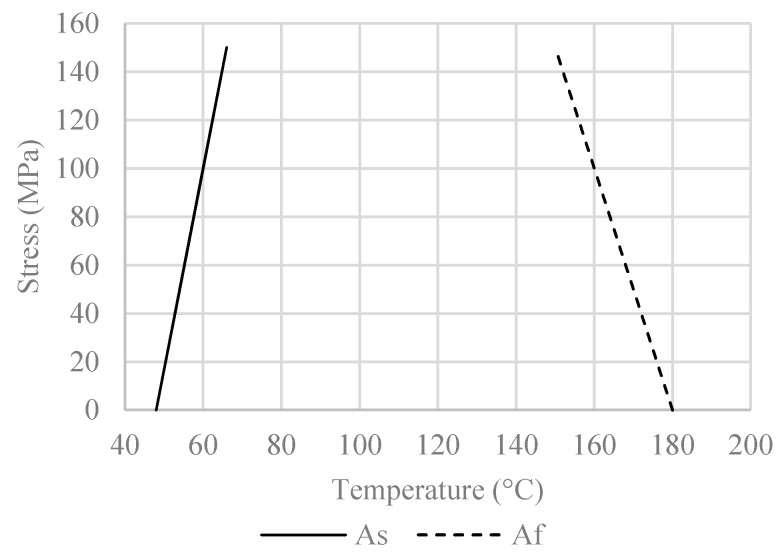
Austenite start and finish range.

The austenite start (As) and finish (Af) threshold values for each phase of the alloy were obtained from experiments conducted by the research group of the Laboratory of Experimental Strength of Materials and Structures, as depicted in Figure 4.

**Figure 5:**
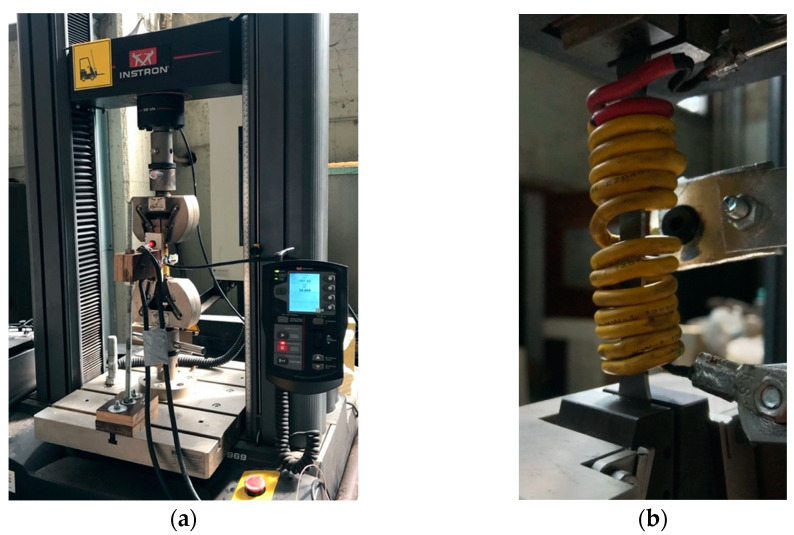
(**a**) Instron 596 tensile testing equipment; and (**b**) specimen heating coil, part of the induction heating machine.

The Instron 596 tensile testing machine was employed for the thermomechanical tests, as shown in Figure 5. The Instron machine contains an integrated dynamic load cell with a capacity of 50 kN, while its operating system is the Bluehill software data acquisition system. During the mechanical loading tests, the strain evolution was monitored using a clip-on extensometer with a gauge length of 50 mm. The thermomechanical tests, which employed displacement control, were monitored through the Bluehill system. The thermal cycles were conducted using a custom-made induction heating and air-cooling machine, which is also shown in Figure 5. The machine was programmed and manufactured internally in the laboratory for the scope of the thermomechanical experiments performed by the research team.

### 2.1. Tensile Test Procedure

During prestressing, SMA tendons are expected to generate compressive stresses, adequate for the purpose of balancing out the impact tensile stresses have on concrete members. SMA tendons can vastly facilitate on-field pre-stressing: being already in a prestressed state, the sole remaining process to be conducted in situ would be heating, which would enable the activation of the shape memory effect; thus, producing the required recovery stresses. SMAs would therefore eliminate many of the common disadvantages that emerge from the mechanical pre-tensioning process.

**Figure 6:**
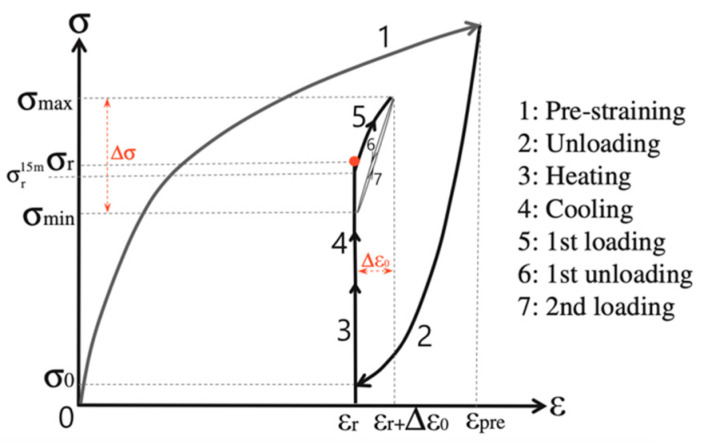
Cyclic loading path [40].

The test profiles were based on the tensile and fatigue tests conducted by Ghafoori et al. [40]. The test profile is presented in Figure 6. Three initial experiments were performed in order to characterize the mechanical properties of the alloy. The yield and ultimate strength and strain were acquired through this process. Strain was monitored using an extensometer and the displacement values were recorded directly through Instron’s software. Simultaneously measuring strain and displacement was crucial in order to record the correlation between the two values and subsequently use it for calibration (this is explained more thoroughly in the following sections).

**Figure 7:**
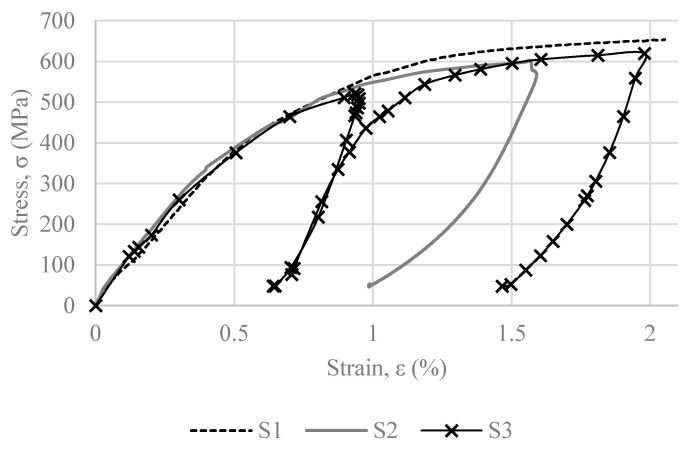
Stress vs. strain curve, specimens S1–S3.

One standard tensile test (S1), along with two loading–unloading sequences with target strains of 2% and 1.6% (S2 and S3), were conducted. Figure 7 presents the tensile tests results. The experiments were performed using strain–control under a strain rate of 0.15%/s, as obtained from the literature [40].

Different target strains were employed throughout the tests, aiming to examine how the thermomechanical response of the alloys varies in relation to this change. Altering the pre-strain values has a practical purpose in civil engineering applications. During on-site prestressing, it is challenging to acquire a high level of precision. Thus, a target pre-strain of 2% might eventually turn out to be 1.6%, 1.8% or 2.2%. Given this fact, the research team decided to employ varying pre-strain values. The concept of the unstable test parameters is further illustrated in the following sections.

**Figure 8:**
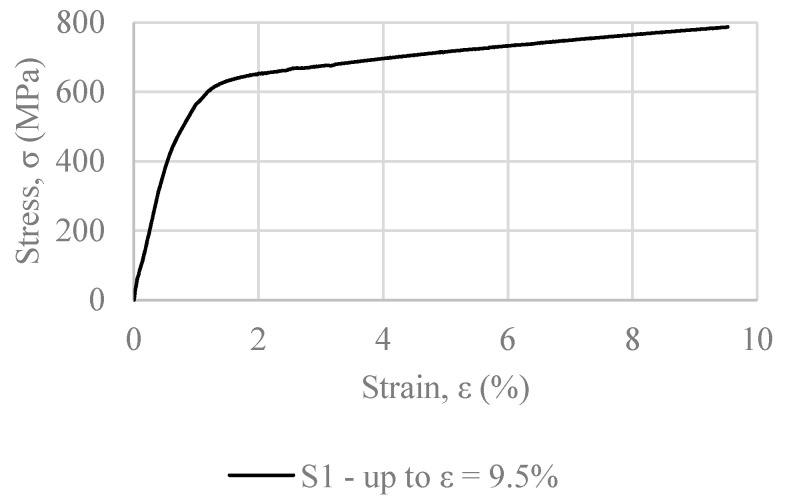
Stress vs. strain curve up to 9.5%, specimen S1.

S1 was the first specimen to be tested. Figure 8 presents the stress vs. strain curve derived from the uniaxial tensile test of S1, up to 9.5% strain. Following this experiment, S2 was tested up to 1.6% strain; and S3 was tested in two increments, firstly up to 1% and subsequently up to 2% strain (see Figure 7).

### 2.2. Low-Cycle Fatigue Test Procedure

The fatigue test profiles and loading protocols were also based on the literature [40]. The aim of this test setup is to investigate the recovery stress generation, while strain is monitored. The applied loading path is presented in Figure 6.

Various researchers have already dealt with strain recovery formation; however, there are few studies referring to the recovery stress. In civil engineering applications, the recovery stress is highly significant since when prestressing a member, the emphasis is given to the maximum stress that a tendon can produce. SMAs offer the possibility of a new prestressing technique that is simpler and easier to conduct on field.

In addition to calculating the amount of recovery stress that can be generated, estimating how well SMAs tendon can perform over time and under cyclic loadings is equally substantial for prestressing applications. The tendon will most likely experience fatigue since it is expected to receive various additional live loads and potentially even seismic loads. Measuring the relaxation that occurs during low-cycle fatigue loadings is, therefore, essential to determine the applicability of SMAs in this configuration.

Three variables that directly influence material behavior on fatigue are considered in the tests: loading frequency, level of pre-strain and limit stress target value. These parameters are variable in each test. The deviations aim to simulate the conditions of on-field prestressing, where the level of precision is most likely lower than in a laboratory setup. Each parameter is briefly highlighted below:

Loading frequency: Four different frequencies are examined: 0.5, 1, 2 and 4 Hz, which are imposed on the samples C1, C2, C3 and C4, respectively.
Level of pre-strain: Pre-strain values are denoted as ε_r_ in Figure 8. For the present study, pre-strain values of 1.85%, 1.80%, 1.90% and 2% were imposed on the specimens C1, C2, C3 and C4, respectively.
Limit stress target value: Thermal expansion, which occurs at the beginning of the heating process, induces stress drop [40]. In order to avoid the formation of compressive stresses during that phase, setting a lower stress limit is practical. While Ghafoori et al. [40] employ a stable lower limit of 50 MPa, the present study uses four different ones: 125, 70, 120 and 150 MPa on samples C1, C2, C3 and C4, respectively.

Due to testing configuration limitations, strain control was not feasible. As can be seen in Figure 5b, the induction heating coil that is used for the thermal cycles poses a practical restriction for the positioning of the extensometer. Similarly, a strain gauge would be useless in such an application since the high temperatures would affect the measurements. Thus, after careful strain-to-displacement calibration was conducted through the tensile tests, displacement control was performed. However, all the results were carefully converted and presented in strain values for the purpose of comparability.

The detailed cyclic test profile loading pattern for all four of the specimens is presented in Figure 8, as obtained from Ghafoori et al. [40]. Both tensile loading and unloading were performed at a strain rate of ε = 0.075%/s. After reaching the target pre-strain, the specimen is unloaded. Both loading and unloading, paths 1 and 2 of Figure 8, respectively, take place at room temperature. The subsequent step (path 3 of Figure 6) is heating. The specimen is heated at 160 °C and then, cooled back to room temperature (path 4). After reaching room temperature, the strain is kept constant for 15 min in order to monitor any potential stress relaxation. According to findings in the literature, [40], this hold time is sufficient for the most part of the relaxation to occur. Stress relaxation is attributed to creep phenomena [40,41].

Following the end of the hold time, 10 low-frequency cycles were performed at the same strain range, 0.04%; however, they were performed at different frequencies. After the cyclic loading, all the specimens were subjected to an increasing tensile load (again, through displacement control), with the ultimate target strains being the initial pre-strain values of each specimen.

## 3. Results

### 3.1. Tensile Tests

The results of the three initial experiments are presented in Figure 7. All three of these tests were performed at room temperature. According to the recorded data, the standard 0.01% and 0.2% strength of the material are 175 and 510 MPa, respectively. For the purposes of this study and in accordance with the literature [40], two limits are investigated: the 0.01% stress, as a limit of proportionality; and the limit stress that corresponds to 0.2% strain. This consideration is further explained in the following section.

S1 was tested first and produced an ultimate tensile strength of 1066 MPa. Specimen S2 was loaded until a pre-strain of 1.6%, which will be a target strain for the cyclic tests. It was observed that the stress recovers from 1.6% to almost 1%. Specimen S3 was loaded in two increments in order to determine if the loading–unloading and reloading sequence influences the stress that corresponds to 2% strain. According to the experiments, there is only a slight deviance of approximately 5%, which is considered negligible. From this experiment, it was also concluded that for a target strain of ε = 1% and ε = 2%, the recovered strain is equal to 0.65% and 1.5%, respectively. It can also be observed that the slope of the curve changes at approximately 300 MPa due to phase transformation.

### 3.2. Low-Cycle Fatigue Tests

**Figure 9:**
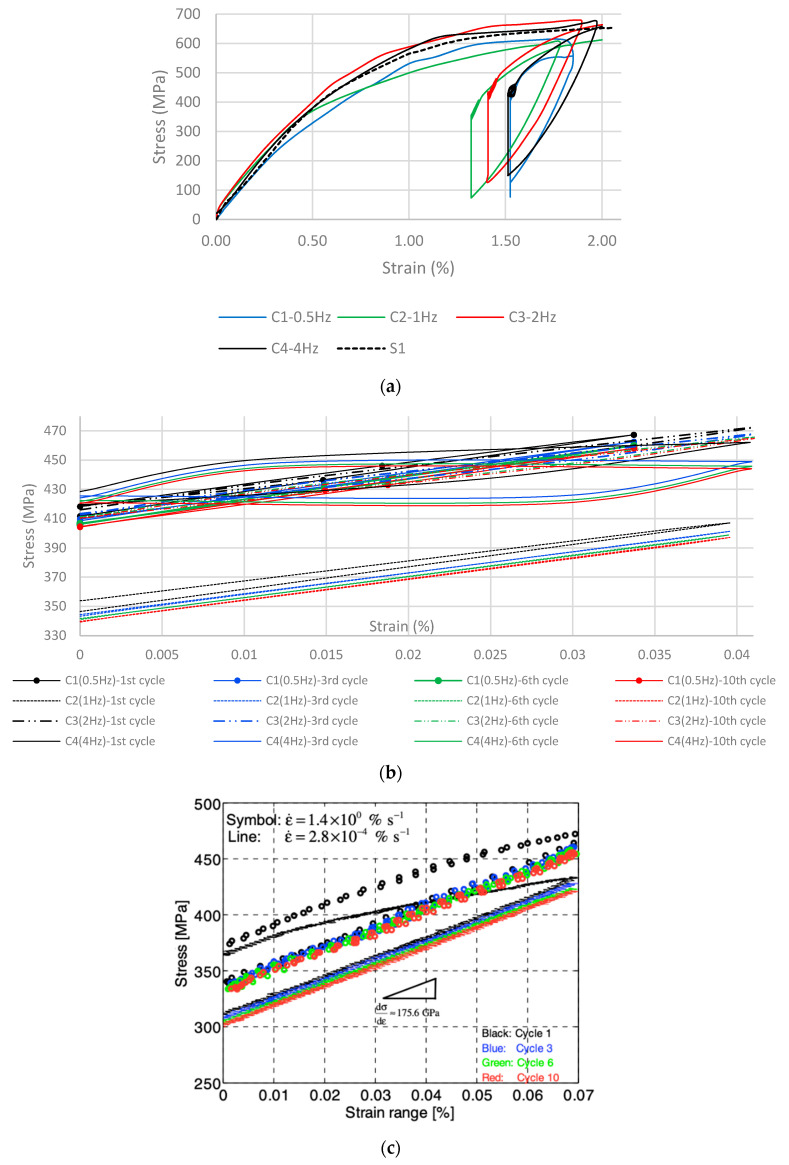
(**a**) Fatigue tests results. (**b**) Detailed graph of the alloy’s cyclic behavior at the programmed strain range. (**c**) Ghafoori et al. [40]: detailed graph of the alloy’s cyclic behavior at the programmed strain range.

The results of the fatigue tests are displayed in Figure 9a–c. Figure 9a is the stress–strain graph for each specimen, plotted with the stress–strain curve of S1, which serves as a reference. After the completion of the loading–unloading path, significant recovery stresses are generated, ranging from 265 to 275 MPa. However, this is attributed to both SME and thermal contraction. Upon heating and until 100 ℃, thermal expansion is prevalent; therefore, the monitored stress drops at the beginning of the thermal cycle. Once this temperature is exceeded, SME becomes the governing factor; thus, stress starts to increase. As soon as the cooling phase is initiated, thermal contraction further increases the stress.

After the thermal cycle is finished, an initial recovery stress is recorded, which is denoted as σ_r_ in Figure 5. The hold time between the thermal and the mechanical loading cycles ensures that stress relaxation is allowed to take place. The subsequent mechanical loading begins from the reduced recovery stress, σ_min_ (see Figure 5). In Figure 9b, it is evident that each frequency has a different effect on the SMA’s behavior.

## 4. Discussion

### 4.1. Tensile Tests

Detecting an exact yield point is a challenging and uncertain procedure that has already been a matter of discussion in the relevant literature. According to Lee et al. [42] and Khalil et al. [43], there are two mechanisms taking place upon loading that complicate the detection of a clear yield stress. Khalil et al. [43] denote two different yield limits, one that occurs due to the activation of stress-induced martensite and another that relates to plasticity. The interaction of these two mechanisms complicates the identification of the precise point where plasticity begins to take effect.

Temperature should also be considered since these two limits are not stable to temperature variations. Additionally, the stress–strain curve’s slope varies at different temperatures. Khalil et al. [43] have produced a series of explanatory graphs and tables that clarify the interaction between the two mechanisms at various temperatures. They report which mechanism is prevalent for each temperature.

According to the literature, the material’s unloading behavior is not linear, as would be expected according to Hooke’s law. The nonlinearity of the unloading curve demonstrates an amount of pseudo-elasticity. The present study’s outcomes are in good agreement with the literature. This behavior is demonstrated by the change of slope in the unloading curves of specimens S2 and S3, presented in Figure 6.

The following paragraphs discuss the major findings of the present research. The adopted loading protocol is chosen from the literature for comparison purposes, taking under consideration the fact that there is a lack of an official testing standard (ASTM or EN) for this type of test (thermomechanical fatigue tests for Fe-SMAs). The investigated parameters are different from the ones reported in the past; thus, they increase the density of the experimental results concerning Fe-SMAs. The different heating rate, loading rate and pre-stress range that are applied are the basic parameters that are discussed hereafter and compared with the literature.

### 4.2. Low-Cycle Fatigue Tests

Table 1 includes all the parameters investigated in each test. It contains details on the pre-strain level, unloading target value, cyclic loading frequency and recovery stress for each specimen. It is expected that different pre-strain and unloading target stresses result in different residual strains and consequently, different recovery stresses. However, the stress drops due to relaxation do not significantly deviate from each other, all ranging between 7 and 9 MPa. The corresponding stress drop percentages are all below 2.10%, which means that the SMA tendons can withstand the anticipated cyclic loads that will be applied on the structure without experiencing major relaxation. After the cyclic loading–unloading sequences, the final recovery stresses are quite high, with increases from the corresponding unloading stresses ranging from 270 to 290 MPa.

Figure 9b displays each specimen’s detailed response on the mechanical cyclic loading–unloading sequence. Figure 9b is presented similarly to the literature [40]. It is evident that between the first and tenth cycle, some amount of the recovery stress was lost. This stress loss is attributed to plasticity. The maximum stress drop is found to occur between the first and the second cycle. This is apparently the stage where plasticity begins to have an effect on the material’s mechanical behavior. However, the stress drop due to plasticity that takes place throughout this process is considered a small fraction of the total stress-drop.

Specimen C4 demonstrates a partly different response to the fatigue loadings compared to C1–C3: It exhibits a clear hysteresis loop for each cycle, as can be seen in both Figure 9b,c. The recorded hysteretic behavior can not entirely be attributed to the increase of frequency since it only occurs for C4 and not for C2 or C3. It can be assumed that the microstructural response of C4 was different from the rest; however, in order to reach a conclusion on the factors that drive this change in behavior, microstructural investigation needs to be conducted. However, the hysteretic behavior of C4 is in good agreement with the literature covering fatigue [22,23,24,25,26,27,28,29,30] since it has been reported that iron-based SMAs have energy-dissipating potential. The hysteresis loop on 4 Hz, thus, verifies the material’s damping capacity. Setting that aside, similarly to the rest of the specimens, C4 demonstrates plasticity mostly during the first cycle; After that, its response is stabilized with each cycle.

**Figure 10:**
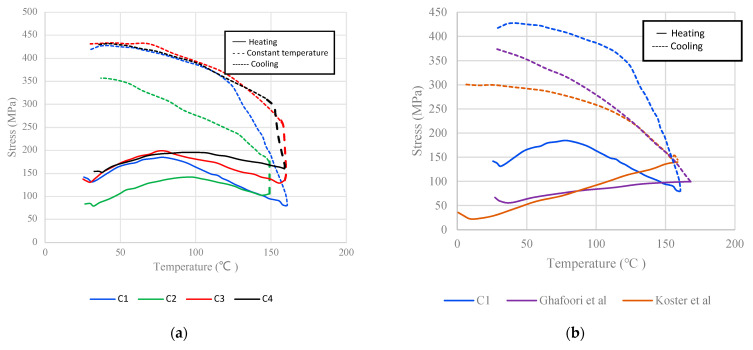
(**a**) Stress vs. temperature graph for all specimens, C1–C4; and (**b**) comparison with the literature [40,44].

**Figure 11:**
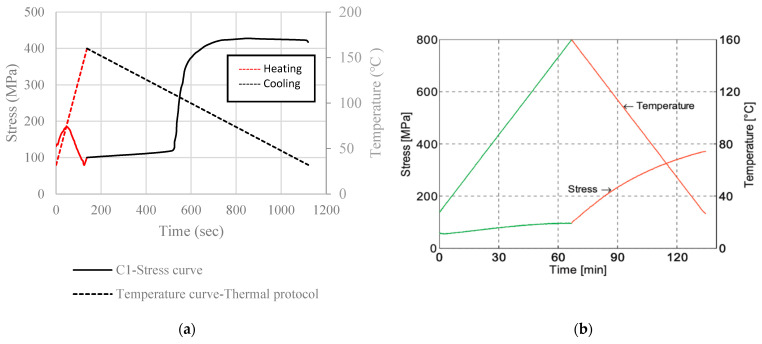
(**a**) Stress–time–temperature graph, specimen C1; and (**b**) stress–time–temperature graph, Ghafoori et al. [40].

Figure 10a presents the stress–temperature curves for the specimens used in the present study. Figure 10b is a comparison between the findings of this study and the literature. The shape of the curves and, therefore, the thermomechanical behavior of the alloy, depends on the heating and cooling rates. It is evident from Figure 10b that the more rapid heating rate induces thermal expansion more intensely than the slow rate does. In this study, the heating and cooling rate is equal to 1.2 °C/s; a very quick heating rate, trying to simulate realistic in situ civil engineering applications (e.g., the increase of temperature that occurs with the use of an inductive current or with a simple flamethrower). On the other hand, the literature adopts a slower heating rate that reaches 2 °C/min [40]. In Figure 10 and Figure 11, the rapid heating rate results in a small initial increase of the recorded stress at the range of 50–80 MPa, followed by an equivalent decrease in stress. During cooling, the shape memory effect (SME) is initialized and coupled with the thermal contraction, it results in the increase of the recorded stress at the range of 450 MPa. Similar findings of the SME initiation and the thermal contraction effect are also mentioned in the literature (see Figure 10b and Figure 11b).

Figure 11a shows the stress–time and temperature–time curves that correspond to the thermal protocol, without the hold time for temperature equalization. The left vertical axis depicts the recorded stress, whereas the right vertical axis the applied temperature. Two curves are presented: the stress curve (continuous line) and the temperature curve (dashed line). The time record upon heating and cooling is different since heating was performed using a standard rate, whereas cooling was completed naturally. In combination with Figure 4, the interaction between the SME and the thermal expansion effect can be better understood: firstly, Figure 4 shows that the austenite start ranges between 50–80 °C. From Figure 10a, it can be seen that stress increase takes place around 40 °C and is continued until 70 °C. Therefore, during the austenite start, stress increase occurs at an almost constant rate and it can be considered that at this point, the SME is dominant. After 70 °C, a rapid decrease in stress is observed; thus, for the temperature range of 70–110 °C, thermal expansion appears to be prevalent. From 110 °C to about 160 °C, an increase is observed at a significantly lower rate. Therefore, coupling of the two effects takes place at this point, with the SME prevailing. During cooling, SME is still present since the quick heating rate prevents the phase transformation from martensite to austenite, to be entirely completed before cooling begins. Namely, it takes some time for the specimen to distribute the temperature changes across its whole volume, which means that the initiation of cooling does not signify the end of the SME. A slower heating rate would allow the temperature to be more uniformly distributed and such peaks would be avoided; an overall smoother curve (similar to the literature’s) would most likely be formed. After 110 °C, a rapid increase in stress is observed due to the thermal contraction effect.

Figure 11a,b demonstrate that the heating–cooling rate plays an important role in the thermomechanical behavior of the alloy, especially in terms of the thermal expansion effect and the alloys’ temperature distribution across its whole volume. The specimens used by Ghafoori et al. [40] were heated at a rate of 2 °C/min, while the samples of the present study were heated at an average rate of 1.2 °C/s. The specimens are left to cool naturally. According to the monitored data, cooling occurs at an average rate of 8 °C/min, (~0.13 °C/s), which is evidently slower than heating.

## 5. Conclusions

The present study focused on the thermomechanical behavior of an iron-based shape memory alloy subjected to tensile and low-cycle fatigue tests. A set of specimens was subjected to a certain target of pre-strain values, thermal and then, mechanical cyclic loading for different frequencies. Under constant strain conditions, the recovered stress was monitored. The following conclusions were drawn from the experimental investigation:

1. Cyclic loading was applied with four different frequencies (i.e., different strain rates) that were missing from the literature. It was shown that while at 0.5, 1 and 2 Hz, the behavior of the alloy was similar, at 4 Hz, a hysteresis loop was observed. This finding confirms the alloy’s energy-dissipating capacity.
2. The studied alloy demonstrated a significant recovery stress, which was largely maintained after the fatigue tests. After the loading cycles, a small drop in the recovery stress, around 2–3% was observed. Moreover, the measured recovery stress decrease, during the 15 min hold time, was relatively small.
3. The experimental results are promising in terms of the on-field prestress feasibility. Recovery stress values are high enough, although accuracy can be an issue that needs careful handling.
4. The heating and cooling rate has a profound effect, significantly changing the thermomechanical behavior of the material.

## Figures and Tables

**Table 1:** Low-cycle fatigue test results.

Specimen	Pre-Strain (%)	Unloading Target Value (MPa)	Residual Strain ε (%)	Recovery Stress σ_r_ (MPa)	Recovery Stress after 15′ σ_r_^15min^ (MPa)
C1	1.85	125	1.50	427	418
C2	1.80	70	1.30	354	347
C3	1.90	120	1.40	424	416
C4	2.00	150	1.50	437	428
**Specimen**	**Stress Drop Due to Relaxation (MPa)**		**Stress Drop Percentage (%)**	**Cyclic Loading Frequency (Hz)**	**Recovery Stress after Cyclic Loading σ_r_ (MPa)**
C1	9		2.10	0.5	404
C2	7		2.00	1	340
C3	8		1.90	2	409
C4	9		2.10	4	420

## Data Availability

The data can be requested by the authors.
